# Museum-based learning for cultural heritage: Examining primary students’ awareness and perceptions

**DOI:** 10.3389/fpsyg.2026.1836754

**Published:** 2026-05-21

**Authors:** Ahmet Akif Erbaş, Leyla Akcan, Emrullah Akcan

**Affiliations:** 1 Department of Child Care and Youth Services, Ahmet Erdoğan Vocational School of Health Services, Zonguldak Bülent Ecevit University, Zonguldak, Türkiye; 2 Independent Researcher, Gaziantep, Türkiye; 3 Department of Primary Education, Faculty of Education, Gaziantep University, Gaziantep, Türkiye

**Keywords:** cultural heritage education, museum-based education, primary school, sustainability education, sustainable cultural heritage

## Abstract

The preservation of cultural heritage and its transmission to future generations is recognized as one of the fundamental dimensions of sustainability. In this context, museums are important learning environments that enable individuals to learn by experiencing cultural heritage. However, it is evident from the literature that there are a limited number of empirical studies that comprehensively examine the effects of museum-based educational practices on students’ academic learning, as well as their perceptions of sustainability and cultural heritage, particularly at the primary school level. This situation highlights the need for further research into how museum-based cultural heritage education contributes to formal educational processes. Building on this gap, the aim of this study is to examine the impact of museum-based cultural heritage education, implemented at the primary school level, on students’ academic achievement and their perceptions of sustainability and cultural heritage. The research was conducted using a mixed-methods approach. A pre-test–post-test control group experimental design was used for the quantitative dimension. For the qualitative dimension, student interviews were conducted to elicit their learning experiences and perceptions. The research findings indicate that museum-based cultural heritage education is an effective teaching approach that enhances students’ academic achievement, fosters awareness of cultural heritage and sustainability, and supports their cognitive and affective development. This study makes a unique empirical contribution to the literature as one of the limited number of studies examining museum-based learning and cultural heritage education at the primary school level using a mixed-methods approach. The findings demonstrate that integrating museum-based learning environments with formal education programs offers significant pedagogical implications for strengthening cultural heritage education.

## 1 Introduction

Museums are institutions where cultural, artistic, scientific, and social values from different periods of human history that have survived to the present day are systematically collected, preserved, researched, and passed on to future generations. The historical roots of the concept of museums emerged as a reflection of the spiritual bond that people established with the visual arts. Over time, museums diversified in parallel with scientific developments, became institutionalized, and acquired universal characteristics (Buyurgan and Mercin, 2005; Öztekin, 2014). In this respect, museums have not remained merely places where historical artefacts are displayed, but have also become living educational environments where learning, cultural transmission, and social memory are reproduced (Giebelhausen, 2006). Today, museums are important learning environments that offer the opportunity to discover different cultures, civilizations, art forms, and scientific developments together (Şar and Sağkol, 2013). Museum visits enable individuals to understand historical context, make cross-cultural comparisons, and relate to artistic aesthetics. Museums nurture children’s curiosity, increase their excitement for learning, and support lasting learning thanks to their environments enriched with visual, auditory, and tactile stimuli. The museum experience, especially for children, offers a powerful learning experience in terms of enabling individuals to become aware of themselves and their surroundings, think critically, ask questions, discuss, and participate in creative processes (Coşkun and Bulduk Türkmen, 2024).

Museums are not merely spaces for displaying artefacts. They also serve as bridges for preserving cultural heritage and passing it on to future generations (Giebelhausen, 2006). In the modern understanding of museology, museums are defined as inclusive, multi-voiced, and critical spaces for dialogue (International Council of Museums, 2025). Redesigning the relationship between museums and society to make them more inclusive, democratic, and multi-voiced spaces is at the center of contemporary museum debates. In this context, museums are being reshaped as laboratories and collaborative spaces where knowledge is co-constructed with society, transcending their traditional boundaries (Facuse Munoz and Cavalcanti, 2024). In this transformation process, museums have become rich educational environments that support lifelong learning (Grack Nelson and Cohn, 2015; Kristinsdottir, 2017).

The paradigm shift in education and the socio-cultural transformation of museums highlight the view that learning is not limited to school walls; rather, it is deepened through experiences in different environments. Out-of-school learning refers to the educational process extending beyond the classroom and taking place in diverse environment based on experience (Bozdoğan, 2016). The inquiry-based learning approach, which is among the key objectives of education, requires the use of out-of-school learning environments that offer students meaningful and lasting learning opportunities (Ministry of National Education, 2018). Museums, historical sites, botanical gardens, and nature are among the fundamental components of out-of-school learning, with museums occupying a particularly important position (Laçin Şimşek, 2020). A review of the literature reveals that out-of-school learning approaches, which extend the educational process beyond the classroom (Bozdoğan, 2016), are closely linked to the lifelong learning model, which ensures continuous individual development. Museums, which have become dynamic and rich educational environments within historical transformation (Grack Nelson and Cohn, 2015; Kristinsdottir, 2017), are located at the intersection of these two concepts. These out-of-school and lifelong learning experiences offered by museums in collaboration with schools are also considered an approach that is directly aligned with the achievement of sustainable education goals (Benuğur, 2000). Museums offer unique opportunities for scientific, artistic, and cultural learning within these environments. Numerous studies exist on the use of museum environments in subjects such as science, social studies, visual arts, and life skills (Aladağ et al., 2014; Atilla and Bulut, 2017; Bozkurt, 2022).

Museum pedagogy is a learning approach that positions students as active learners through engagement with objects, artefacts, and spaces. This active learning process draws its theoretical foundations from Kolb’s Experiential Learning Theory. According to Kolb’s theory, learning occurs when individuals interact with their environment and transform and restructure their concrete experiences through reflective observation and abstract conceptualization. Museum environments are unique experiential learning spaces in this regard. They enable students to establish direct ‘concrete experiences’ with real objects, make historical and cultural inferences through these objects, formulate hypotheses, and develop critical evaluation skills (Dündar, 2003; Tosun, 2015).

This active process, grounded in experiential learning, also plays a critical role in ensuring the cultural sustainability of societies. Preserving cultural heritage, passing it on to future generations, and ensuring continuity in the construction of societal identity are among the fundamental priorities of contemporary education systems. Cultivating individuals who recognize, question, and reinterpret this heritage from a sustainable perspective requires a holistic educational process that begins at an early age. In this context, museums play a strategic role in education as living learning environments that house concrete and unique examples of historical and cultural heritage (Hirzy, 1998).

Museum spaces enable students to relate classroom learning to real life, make historical inferences through objects, and experience cultural context in a multidimensional way (Pasquariello and Maggi, 2003). Museums are an important element of the lifelong learning model, offering voluntary, self-directed, emotional, and intellectual learning opportunities to individuals of all ages. As public pedagogical spaces, museums are places where communities can see their cultural heritage and social and artistic values (Sandlin et al., 2010). UNESCO (2011) does not limit cultural heritage to material assets alone; it defines it within a broad framework that also encompasses oral traditions, rituals, social values, craft practices, and ways of life. This definition reveals that the transmission of cultural heritage, both its tangible and intangible elements, to future generations is a requirement for social sustainability (Smith, 2006; Harrison, 2012).

Cultural sustainability involves the preservation, perpetuation and transmission to future generations of the knowledge, skills, traditions, values and cultural heritage that constitute the identity of societies (Soini and Birkeland, 2014; Gomez Hurtado et al., 2020). Museum education supports students in recognizing their cultural heritage, appreciating cultural diversity and developing respect for different cultures (İlhan and Okvuran, 2001; Sağlamgöncü, 2023). Museums make significant contributions to strengthening cultural heritage awareness, constructing national identity, developing critical thinking skills, and preserving cultural values. Current research shows that high-quality virtual museum experiences also profoundly strengthen individuals’ perception of cultural identity, thus serving as a powerful motivator that directs them towards actual museum visits in the context of cultural heritage participation (Meng and Dolah, 2025; Bian et al., 2026).

This museum pedagogy, which focused on cultural sustainability, offers a vision integrated with the United Nations Sustainable Development Goals. Sustainable education is a holistic approach that encompasses not only environmental but also cultural and social sustainability (UNESCO, 2017). Museums play a central role in both preserving cultural heritage and transmitting it through education, creating a vital learning environment for cultural sustainability, which is one of the fundamental pillars of sustainable education (Pavlou, 2012). The development of systematic and curriculum-based educational activities by museums in collaboration with schools (Benuğur, 2000) facilitates students’ acquisition of cultural heritage values. This directly supports the Sustainable Development Goals’ dimensions of ‘Quality Education’ (Goal 4) and ‘Sustainable Cities and Communities’ (Goal 11). Moreover, it is important for cultural institutions to place innovative practices and multidimensional narratives at the center of their institutional strategies in order to successfully implement sustainability processes and keep social memory alive (Topkan and Erol, 2022). Furthermore, it is emphasized that these organizations must adopt a holistic management approach that simultaneously considers economic, social, environmental, and cultural dimensions (Pop and Borza, 2016).

Local museums are one of the most powerful concrete areas where sustainable development goals, cultural memory, and experiential learning intersect. They have a special function in terms of cultural learning because they enable students to establish a direct relationship with their living environment. Students’ understanding of their city’s cultural heritage fosters comparative cultural awareness and strengthens local identity (Crooke, 2008; Sirivanichkul et al., 2018). In this context, Zonguldak Province represents a strategic center in terms of Türkiye’s coal production and mining history. The city’s economic and social structure has been shaped by mining activities. This has become one of the main elements determining Zonguldak’s cultural memory (Güdü Demirbulat and Saatcı, 2020). Mining is not merely a production activity. It is also a cultural practice that encompasses multidimensional social processes such as a culture of solidarity, risk management, working life, class consciousness, and forms of social organization (Arslan and Kerestecioğlu, 2022).

Cultural heritage refers to the entirety of tangible and intangible elements that a society values and aims to pass on to future generations (Nart, 2015). For museums to function as sustainable cultural destinations, it is necessary to adopt an experience-oriented sustainable cultural management approach that meets visitor expectations (Vargas Sanchez, 2015; Shin et al., 2026). The Zonguldak Mining Museum, which houses one of the most comprehensive collections on Türkiye’s mining history, is also an important cultural heritage destination on the European Route of Industrial Heritage (2025). The museum makes local memory visible by presenting visitors with the historical development of mining technologies, working conditions, production processes, documentary archives on workers’ lives, oral history records, and photo collections. For primary school students, the mining museum offers a unique ‘concrete experience’ environment, as defined by Kolb, which concretizes the relationship between local history and daily life. In this space, students have the opportunity to directly observe local cultural heritage, understand how economic activities shape social life, comprehend the relationship between mining and the concepts of labor, solidarity, and risk, and develop an awareness of conservation in the context of cultural sustainability.

The contribution of museums to individuals’ cognitive, emotional, and social development is widely discussed in the literature. Various studies have shown that students participating in museum education experience increases in critical thinking, problem solving, creative thinking, attitudes towards cultural heritage, academic achievement, and motivation levels (Holmes, 2011; Yeşilbursa, 2011; Kisida et al., 2016; Mujtaba et al., 2018; Boyacı, 2019; Sofuoğlu, 2019; Guo et al., 2022). Furthermore, museums are cultural living spaces that enable individuals to access social memory and contribute to the sustainability of social existence (Ilicak Aydınalp, 2019). Sustainable education aims to develop cultural, environmental, and social responsibility awareness in individuals; to encourage lifelong learning by preserving the link between the past and the future (UNESCO, 2017). It requires students to understand their environment, gain awareness about preserving cultural values and develop conscious attitudes towards social heritage. It is necessary to protect cultural heritage sites that are at high risk, particularly in the face of modern threats such as increasing human pressure and environmental changes. In doing so, it is imperative to go beyond purely technical measures and develop holistic conservation frameworks and social awareness supported by education (Paruch and Porebska, 2026). In this context, the authentic learning environments offered by museums align with both the theoretical and practical dimensions of a sustainable education approach.

**Figure 1:**
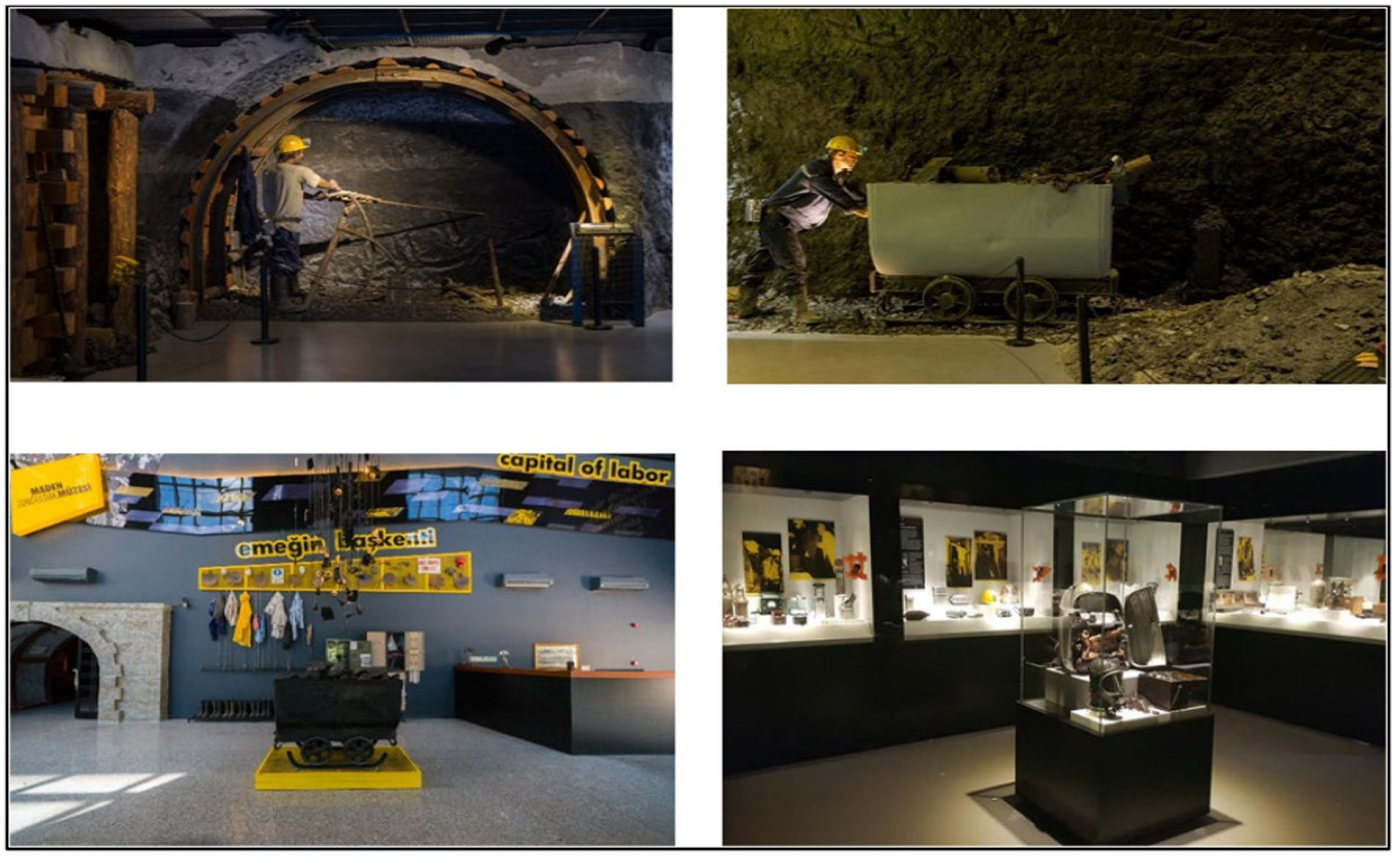
Zonguldak mining museum.

It has been determined that museum visits increase individuals’ levels of interest, curiosity, and excitement (Üztemur et al., 2018; Boyacı, 2019), create long-term cognitive and affective effects, and improve students’ academic performance (Çil and Yanmaz, 2016; Topkan and Erol, 2022). When examining studies on museum education in Türkiye, teacher attitudes and views (Dilli, 2017; Öner Armağan et al., 2023; Çetin and Dağ, 2024), teacher candidates’ experiences (İlhan et al., 2021; Aslan and Gangal, 2023), and student attitudes (Güler, 2011; Şenel Çoruhlu et al., 2024; Yurd and Varnacı Uzun, 2024). However, it is observed that research, particularly at the primary school level (Çil and Yanmaz, 2016; Topkan and Erol, 2022), and studies that address cultural heritage and museum education together in the context of sustainable education are quite limited (Mertoğlu and Gürbey Usta, 2023; Sağlamgöncü, 2023). Based on this gap in the literature, the present study aims to support cultural heritage education through museum education in the context of sustainable education. The study includes activities designed to help students recognize and understand elements of family history, local culture, traditional life practices, economic activities, and social memory within Kolb’s experiential learning cycle. The implementation process was designed within the framework of museum spaces that serve as carriers of urban memory, such as the Zonguldak Mining Museum (Figure 1). Within this framework, the aim was for students to tangibly experience the historical fabric of the city, its mining culture, and the local manifestations of cultural heritage, thereby building lasting awareness in line with sustainable cultural development goals.

The aim of the study is to examine the effects of museum education applications in fourth-grade social studies lessons on student achievement and perceptions. In this context, the study addressed answers to the following questions.

1. Is there a significant difference between the pre-test scores of the experimental and control groups?
2. Is there a significant difference between the post-test scores of the experimental and control groups?
3. What themes shape the experiences of students in the experimental group regarding learning through museums and cultural heritage?

## 2 Methods

This study used a mixed-methods approach, combining quantitative and qualitative methods to enable a deeper understanding of research problems that are too complex to be understood using only quantitative or qualitative data (Creswell, 2014). The quantitative dimension examined the effect of museum-based cultural heritage education on students’ academic achievement, while the qualitative dimension aimed to support the quantitative findings through students’ perceptions, experiences, and awareness regarding the process. In the quantitative dimension, a pre/post-test experimental design was used to examine the effect of the museum-based teaching process on students’ achievement levels. A pre-test was administered to both the experimental and control groups. Subsequently, only the experimental group underwent a six-week intervention program. At the end of the 6-week program, a post-test was administered to both groups.

### 2.1 Study group

The study group for this study consisted of a total of 80 students attending the fourth grade at a state primary school in Zonguldak, Türkiye. The study group was determined using the criterion sampling method (Patton, 2014), where the criteria were:

1. The students had not previously received structured education in a museum environment,
2. They were located in a school district with similar socio-economic characteristics.

Within this scope, the study was conducted in two groups: an experimental group and a control group.

### 2.2 Data collection

In this study, both quantitative and qualitative data collection tools were used. The study was structured within the framework of a mixed-methods approach; with an experimental design used for the quantitative dimension and semi-structured interviews used for the qualitative dimension. Accordingly, two main data collection tools were used; an achievement test and a semi-structured interview form. Parental consent was obtained for all participating students.

To collect quantitative data, was developed a ‘Cultural Heritage Achievement Test’ was developed to measured changes in students’ cognitive levels before and after museum education. Expert opinion was sought to validate the test, and a criterion table was prepared, based on the Year 4 primary school social studies curriculum and textbook. The test initially consisted of 40 questions that addressed each learning outcome. After validation, half the questions were removed, resulting in an achievement test consisted of 20 questions, with each correct answer awarded 5 points, resulting in a total score of 0–100. The reliability analysis of the test resulted in a KR-20 (Kuder–Richardson 20) value of 0.78, which was deemed reliable (Büyüköztürk, 2024).

In the qualitative dimension of the research, a semi-structured interview form to examine the views, experiences, and perceptions of students in the experimental group regarding the museum-based sustainable education intervention. Questions were prepared by reviewing the literature and deepening them with probes (Merriam, 2013). Before implementation, the interview form was reviewed by field experts to ensure its appropriateness in terms of language, comprehensibility, and content validity. Interviews were conducted with students in the experimental group after the intervention program described below.

### 2.3 Implementation process

Our study was based on a six-week training program we developed. After the pre-test achievement test was administered to the experimental and control groups, activities based on experiential learning with the theme of ‘Sustainable Cultural Heritage’ were carried out at the Mining Museum with the students in the experimental group, while the control group continued with classroom teaching within the scope of the existing curriculum. At the end of this intervention (see Table 1 for the schedule and Appendices for example activities), a post-test was administered to both groups, and face-to-face interviews were conducted with students in the experimental group.

**Table 1:** Training program implementation process.

**Sessions** **(40 min, twice/week)**	**Subject of education or learning outcomes**	**Activity**
1. Implementation (Week 1)	Family history research using oral, written, visual sources, and objects.	Introduction to sustainable education and cultural heritage concepts What is cultural heritage? An introduction to Zonguldak’s history and mining culture and its impact on family life.
2. Implementation (Week 2)	Family history research using oral, written, visual sources, and objects.	Family history and social memory Sharing of oral history notes collected by students from miners and miners’ families. A study of cultural continuity and family memory.
3. Implementation (Week 3)	They provide examples by researching elements that reflect the national culture of their family and surroundings. They visit historical sites in their immediate vicinity, such as a museum, mosque, tomb, bridge, madrasa, or caravanserai, or engage in oral history or local history studies.	Preparatory activities for the mining museum Distribution of task cards for objects to be seen in the museum. Why is an object cultural heritage?
4. Implementation (Week 4)	They provide examples by researching elements that reflect the national culture of their family and surroundings. They visit historical sites in their immediate vicinity, such as a museum, mosque, tomb, bridge, madrasa or caravanserai, or engage in oral history or local history studies.	Zonguldak mining museum field trip Observations on the city’s memory and economic life. Drawing pictures related to mining.
5. Implementation (Week 5)	They recognize the main economic activities within their family and immediate surroundings. Using concepts such as income, expenditure, budget, production, distribution, consumption, and occupation, students are encouraged to observe and report on the economic activities within their immediate surroundings.	Sustainable culture workshop Analysis of the museum experience. Generating ideas for sustaining Zonguldak’s cultural identity.
6. Implementation (Week 6)	They recognize the main economic activities within their family and immediate surroundings. Using concepts such as income, expenditure, budget, production, distribution, consumption, and occupation, students are encouraged to observe and report on the economic activities within their immediate surroundings.	Presentation, product display, and evaluation Display of student posters and product portfolios Future Legacy Card writing activity Overall evaluation of the program

### 2.4 Data analysis

We analyzed the quantitative data using the SPSS 22.0 software package. After testing the pre/post-test scores with the Shapiro–Wilk normality test, we compared them using an independent samples t-test for intergroup comparisons with the significance level set at 0.05. For the qualitative data, we identified preliminary themes in line with the interview questions and coded the students’ statements based on meaningful units. To increase reliability, we repeated the coding process and checked for consistency between the codes and themes. Inter-coder reliability was then calculated using the formula proposed Miles and Huberman (1994) with a concordance of over 80%.

## 3 Results

### 3.1 Quantitative findings

Deviations from normality were not significant and values for skewness and kurtosis were within the −1.5 to 1.5 range, indicating that our quantitative data were normally distributed (Table 2).

**Table 2:** Normality test results.

**Points**	**Group**	***N***	**Skewness**	**Kurtosis**	**Shapiro–Wilk**	***p***
Pre-test	Control	40	0.124	−0.451	0.965	0.245
	Experiment	40	−0.231	0.112	0.971	0.312
Post-test	Control	40	0.315	−0.562	0.958	0.189
	Experiment	40	−0.108	0.234	0.982	0.456

The pre-test results indicated that the control group mean was higher than the experimental group mean, but this difference was not statistically significant, indicating that the groups were equivalent at the beginning of the study (Table 3). Following the intervention, the experimental mean was significantly higher than the control mean. Suggesting that the intervention had a large effect (Cohen’s *d* = 1.28) on the achievement levels of participants in the experimental group (Cohen, 1988).

**Table 3:** Results of the independent groups *t*-test applied to determine whether the pre/post-test scores of the experimental and control groups differed.

**Points**	**Group**	***N***	**X**	**ss**	**Sh**_**x**_	**t test**			**Cohen *d***
						***t***	**Sd**	***p***	
Pre-test	Control	40	68.75	17.605	2.784	0.671	78	0.504	−0.15
	Experiment	40	66.25	15.638	2.473				
Post-test	Control	40	66.00	16.494	2.608	5.737	78	0.000	1.28
	Experiment	40	85.13	13.131	2.076				

### 3.2 Qualitative findings

**Figure 2:**
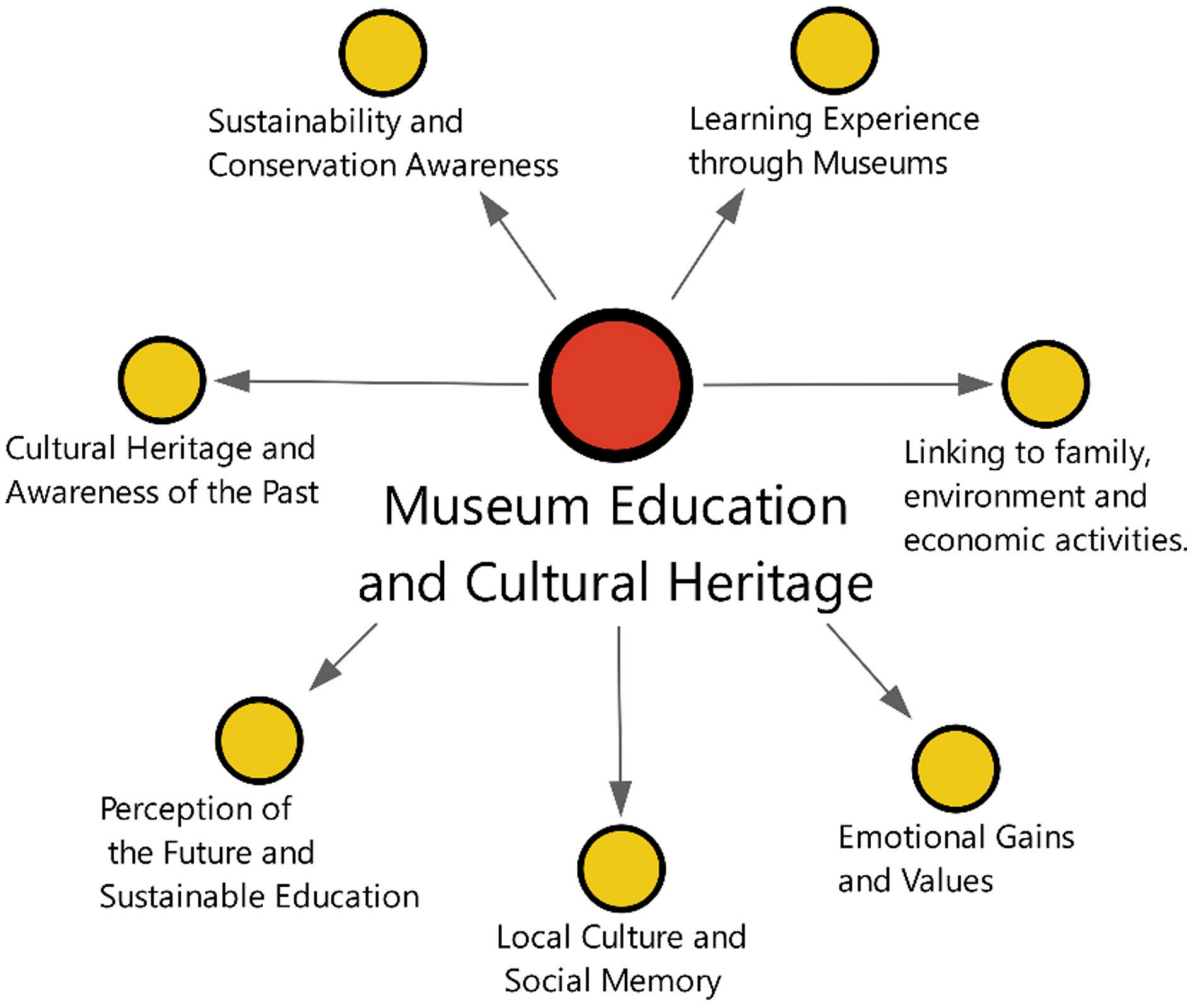
Themes emerging in relation to cultural heritage education.

Interview responses from students in the experimental group were meaningfully clustered under seven specific themes, suggesting that museum-based learning processes have multidimensional and varied effects on students’ levels of awareness, perceptions, and ways of interpreting. The seven themes (Figure 2) imply that museum education is not limited to cognitive learning outcomes.

#### 3.2.1 Cultural heritage and awareness of the past

Students appeared to develop an awareness of the past through their museum experience, relating cultural heritage to past life. Several emphasized that the objects exhibited in the museum facilitated this process, as objects made the past more tangible. In this context, the museum environment was perceived as part of the learning process.

*S1: “The artefacts I saw in the museum helped me understand how people lived in the past. I had seen them in books before. Seeing the items they used made me realize how difficult their work must have been back then. Comparing them to the items we use now, I realized their lives were much harder. But still, those items showed me that people who lived in the past were real. When I got home, I started asking my elders how they used to live.”*

*S3: “When I went to the mining museum, I understood better why my grandfather was a miner. Before, I just thought it was a job, but the tools and photographs I saw there showed me how difficult the work was. When I learned that they worked in dark and narrow places, I began to imagine what my grandfather went through. I gained more respect for him then. I also understood my family’s past better. This trip made me think that professions are not just about earning money, but are part of people’s life stories.”*

*S17: “This museum is like a memory from the past; it must not be lost. Because every object we see there carries a piece of the lives of people from the past. Walking around the museum, I felt as if I had travelled back in time. I thought that if these objects were not preserved, no one in the future would be able to learn about the past. That’s why I realized how important museums are. I think not only adults but also us children should pay attention to these places.”*

*S34: “Seeing the tools used in the past made me feel that the past really happened. I had seen pictures of them in books, but sometimes it seemed like a fantasy. In the museum, I imagined people working with those tools and was very surprised. Especially the things made by hand showed me how patient people were. I could imagine the lives of people living at that time more easily. Thanks to this trip, I realized that history is not just a subject that is talked about, but something that really happened.”*

#### 3.2.2 Sustainability and conservation awareness

Participants stated that the preservation of cultural elements exhibited in museums is important both for today and for future generations, underscoring how museums are necessary for cultural sustainability. Students stated that the museum visit helped them develop an awareness of their responsibility for preservation. In this context, museums have come to the fore as educational environments that support the sustainability of cultural heritage.

*S*7*: “Without museums, old things would be lost, so we must protect them. While walking around the museum, I learned that some of the items are very old, and I thought that if no one had preserved them, we wouldn’t be able to see them today. Then we wouldn’t know anything about the past. Our teacher also told us to be careful when touching them. Now, when I see old things, I try not to break or damage them. Because they are being preserved not only for us, but also for the children who will come after us.”*

*S19: “If these items are preserved without being damaged, the children who come after us can also learn. I was very surprised when I saw the items in the museum because they were all very old but still intact. If people didn’t protect them well, they could have rotted and disappeared. Then, children in the future would not be able to learn about the past. I also thought that I should use my own toys and books more carefully. Because if we protect something, others can also benefit from it and learn new things.”*

*S38: “I learnt that we shouldn’t damage things in the museum. Because they belong to everyone. Before, I just looked around the museum and moved on, but now I understand that the items there are very valuable. If someone breaks or damages something, everyone will be sad, and no one will be able to see it again. That’s why I learnt that we shouldn’t run around or talk loudly in the museum. Now, when I go somewhere, I pay more attention to shared items.”*

*S39: “Preserving the past felt like preserving the future to me. Because if we know what was done in the past, we can do better things in the future. The old objects I saw in the museum showed me how people used to live. If we hadn’t preserved them, we might have made the same mistakes again. That’s why I thought it was important to protect historical artefacts. In the years to come, I want other children to see the things we saw. I learned that historical sites should not be damaged.”*

#### 3.2.3 Learning experience through museums

Some students found the learning process in a museum environment more meaningful than in a classroom setting, as learning by seeing and experiencing enhances the retention of information. Given that museum-based learning is often designed to support active participation and stimulate curiosity, several participants noted that this makes learning more understandable and enjoyable. The museum environment appears to be an effective learning space that enables abstract information to be linked to concrete experiences.

*S2: “I learnt better than I did in class. Because I saw everything here. In the book, we only looked at pictures, but when I saw the real objects in the museum, I understood the subject better. I really enjoyed looking around while our teacher was explaining. For example, seeing an old tool up close made me think about how people worked. Sometimes I get bored in class, but in the museum I never lost focus because I was constantly discovering something new. That’s why what I learned stuck in my mind more clearly.”*

*S9: “Learning in the museum wasn’t like a lesson, it was more fun. Because we didn’t just sit and listen, we learned by walking around and looking. Our teacher asked questions while we were walking around with my friends, and we tried to guess the answers. It made me feel like we were playing a game. Sometimes time passes slowly in class, but I didn’t realize how time passed at the museum. That’s why I remember what I learned at the museum better.”*

*S16: “Learning by touching and looking stuck in my mind more. I can forget some things just by listening, but when I looked closely at some things in the museum and examined them, I understood them better. Seeing the details in particular made me think about how those objects were used. At that moment, I felt like I was someone living in the past. What our teacher told us also came to life in my mind. When I got home, I told my family all about everything I saw at length.”*

*S20: “The museum made me feel like I was inside a book. It was as if the pictures in my textbook had come to life and become real. Standing next to the objects, I imagined their stories. That’s why I felt like I was on an adventure rather than studying. Seeing that the things I read in the book were real surprised me a lot. Learning this way makes it easier for me to remember the topics.”*

#### 3.2.4 Local culture and social memory

Museums served an important function in terms of cultural transmission and sustainability. Students did not evaluate the Zonguldak Mining Museum solely as an exhibition space. They defined the museum as a place that reflects the historical development and economic structure of the city, adding to their historical awareness of their surroundings. In this context, they evaluated the museum as a space of social memory that contributes to their understanding of their city and its past.

*S26: “This museum tells the story of our city. When I entered, I felt as if I had travelled back in time to Zonguldak’s past. The photographs and old artefacts on the walls showed how the people who lived here worked and lived. I understood better why our city is the way it is. Before, it was just a place I lived in, but now it feels like a special city with a past.”*

*S29: “Now I understand why Zonguldak is associated with mining. My elders used to say it, but I didn’t fully grasp what it meant. The tools and tunnel models I saw in the museum showed me how important mining is. I realized that most people in our city have a connection to this industry. Now, when I hear Zonguldak, I think not only of my home, but also of the miners’ labor. That’s why I’ve started to feel a different connection to my city.”*

*S32: “The objects here are like the memory of our city. Because every object has a story. They carry the traces of the people who lived in the past. Without these objects, it would be difficult to understand what our city was like. Walking around the museum, I felt as if I was meeting people from the past. Our teacher also said that these are our past. That’s why I think of the museum as a place that preserves the memories of our city.”*

*S36: “Without this museum, our city’s past could have been forgotten. Because people may not remember everything over time, but everything is preserved in the museum. Seeing the old photographs and artefacts, I learned about the lives of the people who lived here. If it weren’t for the museum, we might never have known this information. I found it very interesting to understand how our city developed. That’s why I think museums are very important and should exist in every city.”*

#### 3.2.5 Linking to family, environment and economic activities

Some students related the knowledge they acquired during their museum experience to their family life and the economic structure of the city. They emphasized that mining has an important place both in their family history and in urban socio-economic development. Several stated that the objects and narratives encountered in the museum corresponded with the experiences heard from family elders, showing that the learning process is interpreted in a personal and social context, and how museum-based learning contributes to the integration of academic knowledge into daily experiences.

*S4: “Since I have family members who worked in the mines, the museum felt more familiar to me. My grandfather and uncle worked in the mines. When I saw the things they talked about in the museum, it was as if their stories came to life. Seeing some of the tools reminded me of my grandfather’s words. That’s why the museum didn’t feel foreign to me. I understood the difficulties my family faced better. I started to respect them more.”*

*S18: “What I saw in the museum was the same as what my family had told me. At home, my elders used to tell me how they worked in the past, but I couldn’t picture it in my mind. When I saw those objects and photographs in the museum, I realized that what they had told me was true. It was as if I had seen their past up close. When I got home, I told them what I had seen and we talked about it again together.”*

*S22: “Here, I learned how people used to make a living. I learned that people earned money by working in the mines and provided for their families. I realized how difficult things were back then. Now I think some things are easier. What I saw at the museum showed me that people lived by working very hard. That’s why I understood better how important it is to work and put in effort.”*

*S30: “I realized why mining is important for our city at the museum. I had previously thought of it as just a job. I learned that it contributes to the development of our city. I learned that people sustain their livelihoods through this work. The stories told at the museum showed me that mining is not just a profession, but a part of our city. I have now started to think more about our city’s past.”*

#### 3.2.6 Emotional gains and values

Participant statements revealed that the museum experience is not solely about cognitive gains; it also fosters emotions such as empathy, respect, and solidarity. Students became aware of the miners’ difficult working conditions and lives, strengthening emotional awareness and value-based thinking. The visuals and objects in the museum environment enabled students to evaluate historical events from a human perspective, helping them develop a more sensitive perspective towards individuals who worked in the past.

*S15: “When I saw that miners worked in very difficult conditions, I respected them. When I learned that they worked in dark and narrow places, I put myself in their shoes. I thought they were very tired and did dangerous work. That’s why I realized how brave they were. I didn’t know it was that hard before. Now I think I need to be more careful and respectful towards people who work hard.”*

*S19: “This museum taught me how important it is to work hard. I learned that people work long hours and struggle for their families. The photos I saw in the museum affected me deeply because everyone looked so tired. That’s when I realized you have to work to achieve something. I now understand that I need to be more patient in my own work. I realized that valuable things come from hard work.”*

*S27: “I felt that I needed to be more sensitive towards people who worked in the past. Seeing the difficulties they faced really affected me. When I thought about how hard people worked back then, I wanted to thank them. I got emotional in some places while walking around the museum because I understood that their lives weren’t easy. Now I see people who lived in the past not just as history, but as real people.”*

*S37: “When I left the museum, I thought I wanted to protect the past. Because I understood that the objects and stories there were very valuable. I thought that if no one took care of them, they could all be lost. While walking around, I felt both happy and a little sad. Because people in the past went through very difficult things. That’s why, when I grow up, I want to do something to prevent historical places from being damaged. I felt that preserving the past is important.”*

#### 3.2.7 Perception of the future and sustainable education

Students did not view museum education solely as a learning process focused on the past, but one that included a sense of responsibility for the future. They emphasized that the preservation and sustainable transmission of cultural heritage is related to the learning rights of future generations. Through the museum experience, they developed a sense of individual responsibility for the preservation of historical values, as well as a desire to contribute to the preservation of cultural heritage in the future. In this context, museum education provides a learning environment that supports students’ future-oriented thinking skills and sustainability awareness.

*S6: “This museum should be preserved for the future. Because everyone should learn. We learned today, but children who come after us should see the same things. If there is no museum, it will be difficult to learn about the past. While walking around the museum, I wanted my little brother to see the things I learned. That’s why I thought museums should be preserved. It’s great that there will be places where everyone can come and learn in the future.”*

*S11: “We learned, and those who come after us should learn too. Because the information we learned doesn’t belong only to us. Other children should also see the things we saw in the museum so that they don’t forget the past. If we protect it, they can also come and visit. Our teacher said this is our responsibility. That’s why I thought we should pay attention to both museums and historical sites.”*

*S34: “Museums exist not only for the past, but also for the future. The artefacts I saw in the museum showed me how people lived. If these artefacts are not preserved, future generations will know nothing. That is why I understand that museums are important for teaching the past to new generations.”*

*S38: “In the future, I would like to protect such artefacts too. Because what I saw in the museum taught me a lot. I thought that if no one protects them, these artefacts could be lost. Perhaps I will become someone who works in a museum. What I learned in the museum made me feel a sense of responsibility. I thought that protecting the past is a beautiful thing.”*

## 4 Conclusion and discussion

When the quantitative and qualitative findings are evaluated together, museum education appears to significantly increase students’ cognitive gains. At the same time, higher-level concepts such as cultural heritage, sustainability, local identity, and future responsibility are supported. The integration of cognitive gains and socio-cultural values in this way demonstrates that the concept of sustainability cannot be addressed solely as an environmental theme. It is also a holistic phenomenon that encompasses social memory, in accordance with current discussions on the need to consider an interdisciplinary approach (Lyon, 2023).

Quantitative results show that the experimental and control groups were equivalent in terms of academic achievement levels before the application, while afterwards, a statistically significant difference favored the experimental group. This shows that the museum-based teaching process is effective in increasing students’ academic achievements related to cultural heritage. The findings are consistent with previous results indicating that museum education improves students’ academic achievement (Holmes, 2011; Çil and Yanmaz, 2016; Kisida et al., 2016; Topkan and Erol, 2022). Supporting abstract concepts with concrete objects facilitates meaningful and lasting learning.

The qualitative results support the quantitative findings and reveal the effects of museum education. Statements regarding cultural heritage and historical awareness indicating that the museum experience provides a sense of authenticity. Students’ ability to imagine the past through real objects and connect with their family history aligns with the active meaning-making processes predicted by constructivist learning theory (Hein, 1995; Dündar, 2003; Tosun, 2015), and this perception helps deepen their attachment to cultural heritage. The literature strongly emphasizes that high-quality virtual/digital museum experiences serve as a motivating factor for physical museum visits through powerful identity construction (Meng and Dolah, 2025).

Students perceive the issue of heritage preservation and conservation awareness not only as a responsibility for today but also as an ethical responsibility related to the learning rights of future generations. This result reveals that museum education has a strong intersection with the concept of sustainable education. The fact that students express that “preserving the past means preserving the future” is important in terms of showing that cultural sustainability awareness can be developed at an early age (Soini and Birkeland, 2014; UNESCO, 2017). Current literature argues that the protection of cultural heritage sites against modern threats such as increasing human pressure and climate change cannot be achieved solely through structural and technical standards. Moreover, this awareness of protection can only be supported by a holistic social protection vision instilled at an early age, as demonstrated in this study (Bian et al., 2026; Paruch and Porebska, 2026).

The theme of learning through museums shows that students perceive the museum environment as a more engaging, enjoyable, and lasting learning space compared to the traditional classroom setting. The emphasis on learning by seeing, examining, and experiencing is consistent with studies showing that out-of-school learning environments positively affect students’ motivation and attitudes toward learning (Laçin Şimşek, 2020; Boyacı, 2019; Bozdoğan, 2016; Erbaş, 2025). This finding demonstrates that museum education can be central to the teaching process.

Students perceive the Zonguldak Mining Museum as a learning space that embodies the city’s history, economic structure, and social memory. Students’ development of awareness of the city in which they live in through their museum experience supports the role of local museums in the construction of cultural identity (Crooke, 2008). This finding demonstrates that museums are of strategic importance not only for national but also for local cultural heritage transmission. It also aligns with current literature, where preserving cultural heritage sites and integrating them into social life and educational processes is one of the most fundamental strategies for strengthening sustainable regional development and local identity (Dudkiewicz Pietrzyk et al., 2026). Furthermore, industrial heritage sites, which are highly fragile and vulnerable to anthropogenic threats, must be reliably transferred to the future. To achieve this, it is necessary to go beyond standard conservation measures. This research confirms that the vital and educational ties established by local communities and younger generations with these spaces form a strong protection mechanism (Paruch and Porebska, 2026).

The theme of linkages between family, the environment, and economic activities demonstrates that museum-based learning enables students to integrate academic knowledge with their personal life experiences. In particular, the overlap between mining culture and family histories strengthens the emotional and social dimensions of learning. This reveals that museum education develops not only students’ knowledge levels but also their values, empathy, and social awareness (Sandlin et al., 2010; Ilicak Aydınalp, 2019). Recent studies confirm that the emotional bonds students form with their past and their strengthened sense of cultural identity are the most fundamental factors determining sustainable participation in cultural heritage and long-term physical visit intentions to museums (Meng and Dolah, 2025).

Overall, our results show that museum-based cultural heritage education provides effective, meaningful, and multidimensional learning outcomes at the elementary school level. Museum education offers a powerful learning environment for developing cultural heritage awareness, sustainability awareness, local identity perception, and future responsibility, in addition to improving students’ academic achievement. In this respect, museum education can be considered a pedagogical approach consistent with sustainability education goals. Current literature states that for sustainability-focused practices and educational strategies to truly succeed, an interdisciplinary approach linking history, culture, and social areas must form the basis of sustainability goals (Lyon, 2023). As out-of-school learning environments, museums extend the educational process beyond classroom boundaries. They not only create momentary awareness in individuals that extends beyond the school period but also help ensure the sustainability of cultural heritage. This is achieved by preserving the link between the past and the future and promoting lifelong learning. When considered in the context of sustainability, the active out-of-school learning process that takes place in museums validates the main objectives of the article by transforming academic knowledge into a lifelong awareness of cultural preservation and a vision of social sustainability.

## 5 Recommendations of the study

It is recommended that museum-based learning activities be integrated into curricula in a more systematic manner, with museums are considered an effective part of the learning process. Strengthening cooperation between schools and local museums is important, so local museums can be used more effectively to help students understand their environment and cultural identity. Current literature emphasizes that museums’ active use of digital innovations is one of the most effective and innovative strategies supporting the preservation of cultural heritage and the promotion of sustainability (Kacerauskas and Schinello, 2025). Therefore, we recommend that museum applications for educational purposes and out-of-school learning environments be supported by digital technologies in the future. Future research is also required to examine the effects of museum-based learning across different age groups and disciplines.

## 6 Limitations of the study

The study was conducted with students attending a state primary school in Zonguldak province, Türkiye. This limits the generalizability of the findings to different regions, age groups, and museum types. The findings are also limited to the perceptions and expressions of the students. Furthermore, as the implementation period was limited to a specific time frame, the long-term effects of museum-based education could not be examined within the scope of this study. Additionally, the fact that the study did not include the views of teachers and parents can be considered a factor limiting the evaluation of museum education from the perspective of different stakeholders.

## Data Availability

The raw data supporting the conclusions of this article will be made available by the authors, without undue reservation.
