# Supplementary material for: Museum-based learning for cultural heritage: Examining primary students’ awareness and perceptions
Source: Front Psychol. 2026 May 21;17:1836754. doi: 10.3389/fpsyg.2026.1836754 (PMC13233227; doi:10.3389/fpsyg.2026.1836754)
Supplement: Supplementary file 1 [file Supplementary_file_1.docx]

Supplementary Material

**Appendix 1.**

**Students' Drawings Related to Mining and Coal Mines**

| **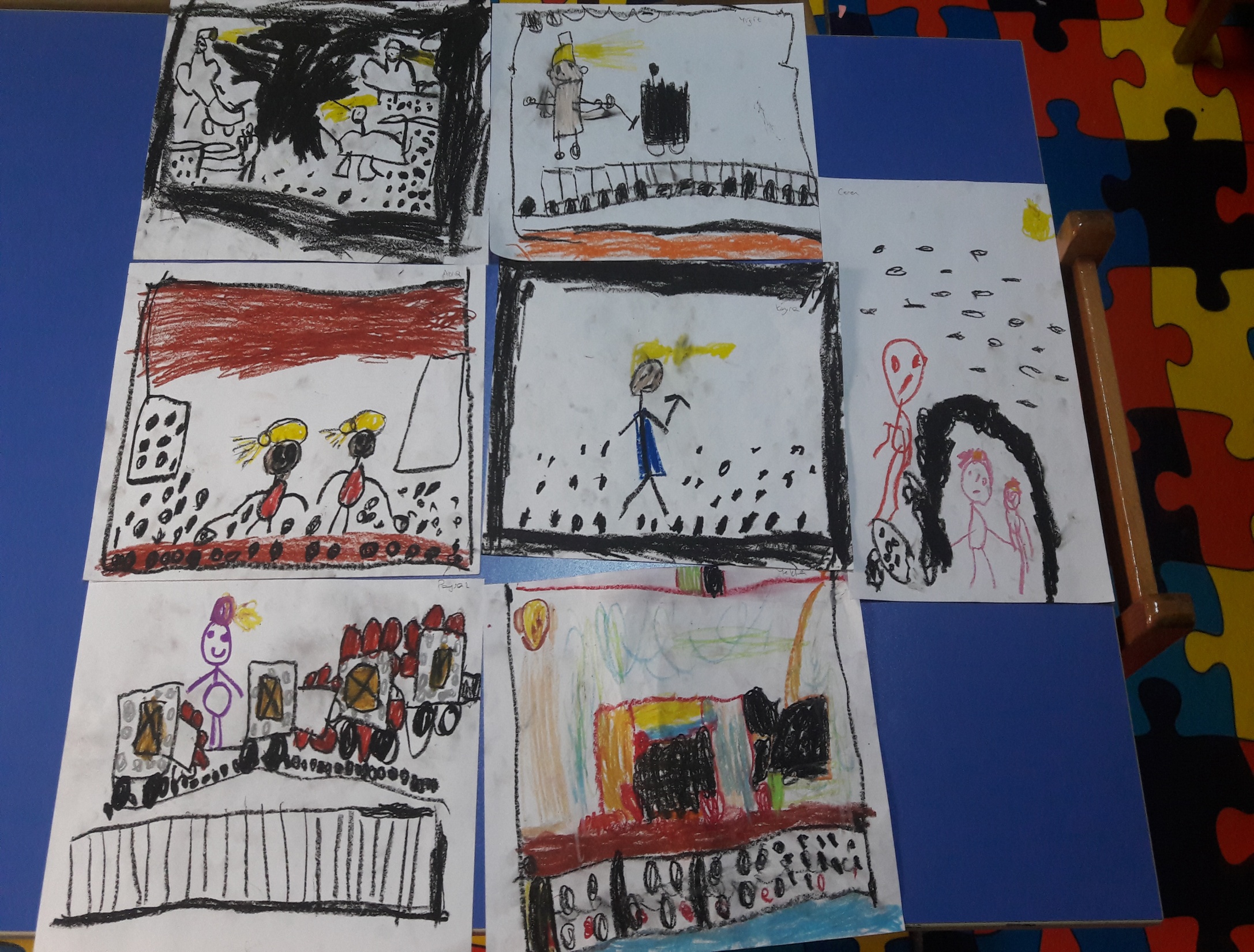** | 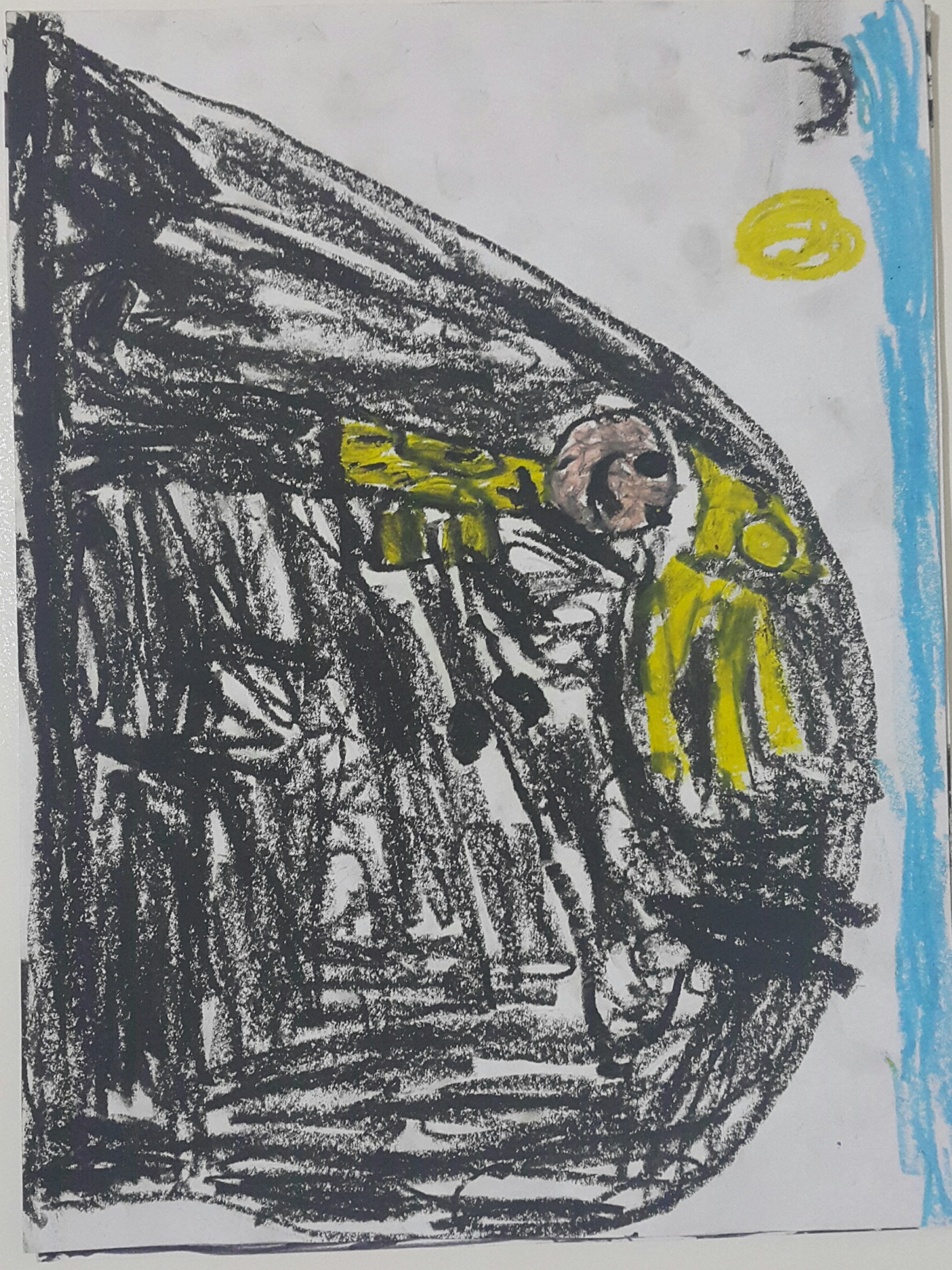 |
| --- | --- |
| **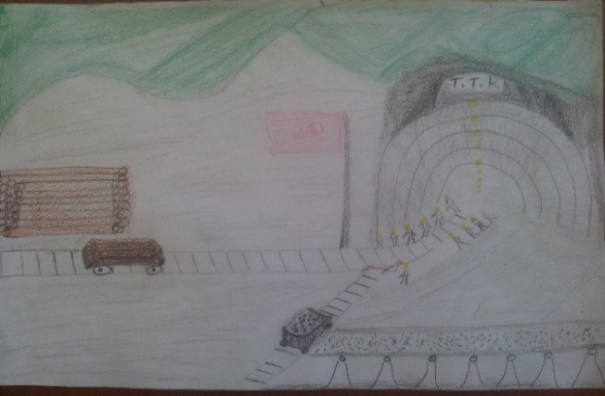** | **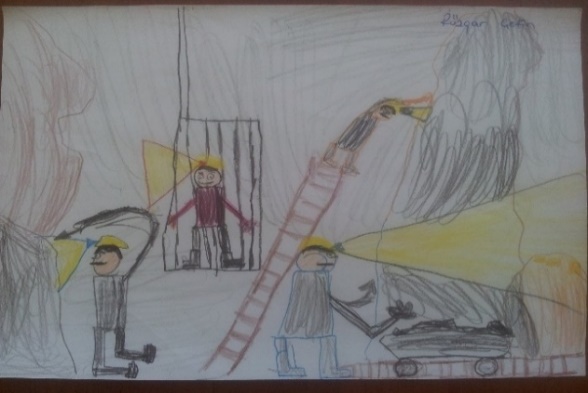** |
| **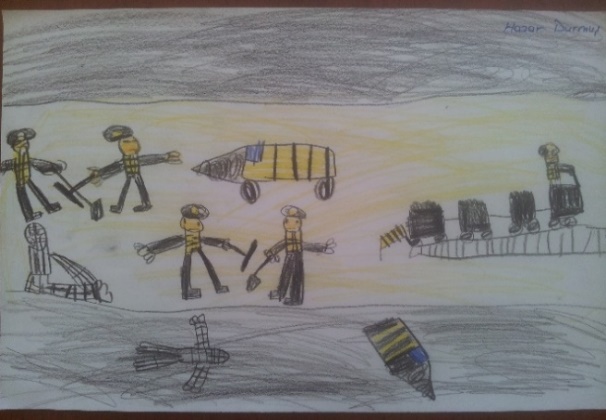** | **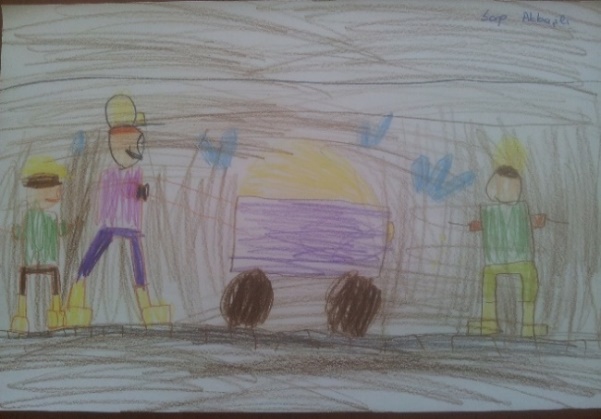** |
| **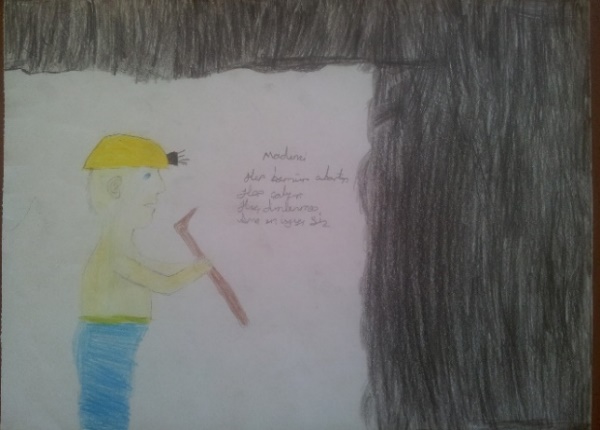** | **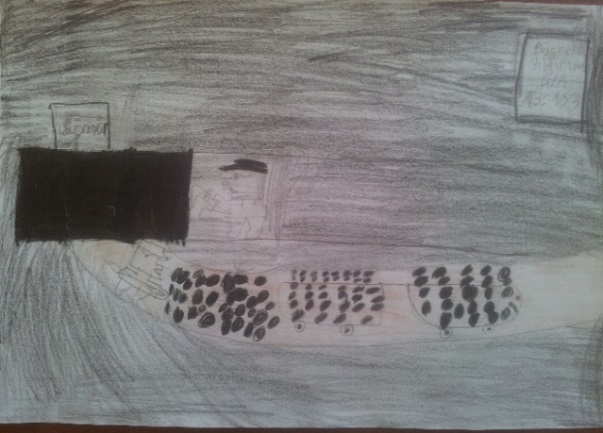** |

**Appendix 2**

**Sample Interview with a Miner and His Family**

**Miner's Name: T.O. (38 years old)**

**A Day in the Life of a Miner:** *Every day, I feel the fear of not returning home safely, of not being able to see my family. Of course, I feel a little scared when I first go underground, but once we start work, there's no chance of thinking about anything else. Because you have to be careful and focus only on the job. Otherwise, there's a high chance of an accident.*

**A Memory of the Miner:** *One day, as I was entering the mine, I felt that today was different from other days. I didn't want to go to work at all, but I had to, so I went. I got ready to go into the mine, then got on the elevator with my friends. It was a 150-person elevator. After going down about 50 meters, the elevator suddenly stopped. We were stuck there for exactly 15 minutes. It's terrifying to think about, but I lived through it. 150 people were stuck in an elevator 50 meters underground for 15 minutes. I experienced fear; I can't even describe the anxiety of wondering if I would be able to get out. After 15 minutes, the elevator started working again and pulled us up. The first moment I stepped outside, I looked up at the sky and said, “Thank God, the world still exists,” and sat down on the side. All my friends took a break for about an hour, and after the elevator was checked, we got back in and went down again. We had to, because we had to do this job to earn our bread.*

**A Day in the Life of a Miner's Wife:** *After my husband leaves home, a sadness comes over me. He works for us. But he's really trying to earn money under difficult conditions. I'm afraid something will happen to him, that he'll have an accident. Every time my phone rings, I answer it fearfully, wondering if there's a problem with him. Until he comes home, I cook meals for him and wait with my children.*

**A Day in the Life of a Miner's Child:** *I feel sad when my dad goes to work. I'm afraid something will happen to him while he's working in the mine. When he comes home, he's very tired. After he takes a shower and changes his clothes, he comes to the kitchen. My mother and I immediately serve him his dinner and tea. He rests in the evening and watches TV. The next day, he goes to work again. To be honest, my brother and I miss him a lot until evening. Even though we want to spend more time with him, I know he works for us.*
